# The Case for Computational Health Science

**DOI:** 10.1007/s41666-018-0024-y

**Published:** 2018-05-30

**Authors:** M. Barnes, C. Hanson, C. Giraud-Carrier

**Affiliations:** 1 Department of Public Health, Brigham Young University, Provo, UT 84602 USA; 2 Department of Computer Science, Brigham Young University, Provo, UT 84602 USA

## Abstract

In this introductory paper, we begin by making the case for Computational Health Science, which we define as the interdisciplinary efforts of health scientists, computer scientists, engineers, psychologists, and other social scientists, to conduct innovative research that will inform future practice directed at changing health behavior through improved communication, networking, and social capital. We recognize and discuss some of the main challenges involved with such an enterprise, but also highlight the associated benefits, which, we argue, significantly outweigh them. We then provide a brief summary of the contributions to this first Special Issue on Computational Health Science.

## Introduction

There have been and continue to be many instances of collaborations across the health and computing sciences [19]. However, in most cases, these are examples of what may be regarded as multidisciplinary activities, where one group may take advantage of the expertise of another, but with only limited, if any, engagement in that other group’s expertise and research agenda [12]. For example, computer scientists may consider health as an interesting area of application, engage health scientists in identifying relevant questions and data, and use health-related data to test their algorithms (e.g., test a novel agent-based modeling algorithm on a known epidemic). Conversely, health scientists may find themselves confronted with a problem requiring the collection of large amounts of data, engage computer scientists in writing the necessary programs, and use the collected data for their own analyses (e.g., utilize big data sources from two health organizations to address public health or health care system needs). In fact, a recent call to action from public health encourages the integration of computer science into public health course work [36]. There is certainly value in such endeavors. However, our vision goes far beyond. While we do not intend to discourage these multidisciplinary efforts, we wish to foster a more truly interdisciplinary approach, one in which we deliberately create, maintain, and pursue joint research agendas that allow meaningful contributions to both disciplines [12].

Computational Health Science (CHS), as we define it, represents the interdisciplinary application of innovative computer science tools, including social network analysis and data mining, to address health-related questions and problems. It integrates the analytical strengths and skills of health scientists and computer scientists, supported by complementary expertise from other researchers and practitioners as applicable (e.g., psychologists, sociologists). The case for CHS includes a number of team learning opportunities arising from the post digital revolution where technology is more ubiquitous. It also calls for learning how to “think together,” as a fundamental ingredient for success in solving complex problems.

This paper serves as an introduction to this Special Issue on Computational Health Science. In addition to providing a brief summary of each of the selected contributions, we discuss the unique opportunities for team learning offered by CHS, as well as some practical considerations to enable successful implementation.

## Team Learning Opportunities in CHS

While creating a deliberate CHS environment comes at a cost, the transfer of technology across the disciplines is likely to occur systematically over time, and with it, significant new opportunities and benefits will be created. Indeed, we argue that, as in all synergistic activities, the whole is greater than the sum of its parts, allowing CHS to do more than could be done in isolation by the constituent disciplines. We outline a few CHS opportunities for team learning and synergism as health/social science and computer science merge to address complex problems.

### Social Media and Public Health Surveillance

Recognizing the usefulness of online data for public health-related purposes, researchers have become more engaged in using computational modeling to better understand health and health behavior. Indeed, computer science expertise is essential for mining large amounts of online information [48]. Pentland et al. [45] have noted that users make daily digital transactions through their use of technology. These transactions “leave digital breadcrumbs – tiny records of our daily experiences” that when mined and analyzed can provide insight into health behavior and health outcomes.

As part of the new twenty-first-century Internet or Web 2.0, social media applications have helped to engage, connect, and mobilize individuals as they freely interact in online communities. Associated applications such as Facebook, Twitter, and YouTube provide the mechanism for organizing individuals into online communities where content can be shared. While some authors have expressed concerns about the use of online and social media data in public health [17], an increasing number of researchers have been quick to point out the novel opportunities offered by these novel data sources to complement, and in some cases, even partially replace, existing practices in health administration, communication, and surveillance, and a number of recent studies have demonstrated the value of online information in understanding public health problems and their determinants (e.g., see [13, 15, 21, 22, 31, 33, 35, 39, 43, 44, 47]).

Specifically, social media applications such as Facebook, Twitter, and YouTube have helped engage, connect, and mobilize individuals as they freely interact and share content in online communities. Recognizing the wealth of information generated by users through their participation with social media, researchers have begun mining this information to gain a better understanding of health outcomes and even health behavior. Several studies have mined YouTube content for information relative to anti-smoking video communities [7], immunizations [34], influenza pandemic [42], quitting smoking [3], cardiopulmonary resuscitation [40], kidney stones [51], and prostate cancer [52]. Similarly, a number of studies have mined Twitter to understand problem drinking [53], detect flu epidemics [1, 2, 11], classify dental pain messages [8, 27], predict depression [14], track suicide [6, 32], gain insight into prescription drug abuse [10, 25, 26], and predict heart disease mortality [16].

### Improved Intervention via Data Fusion

Relevant health data is available from a number of different sources, including traditional ones, such as questionnaires (e.g., NHANES, BFSSR), electronic health records (EHRs), and results of randomized control trials (RCTs), as well as less conventional ones, such as social media interactions, wearable devices, smart homes, and Internet of Things (IoT). While health science has generally focused on traditional data sources, much can be gained by fusing data across many sources to improve intervention and outcomes.

As pointed out by Hesse et al. [28], “research must become rapid if it is to be responsive and relevant to those making treatment and policy decisions now, not 7 to 14 years from now; and more rapid research reduces the risk of producing findings on techniques and procedures that could be dated or obsolete by the time the findings are made available.” Furthermore, they add, “RCTs may be an optimal method for testing the efficacy of a new intervention, but questions such as the effectiveness of the intervention among real patients in real settings, the safety and side effects of the intervention, and the determination of for whom the intervention may be most effective are questions that are better addressed by leveraging health system EHRs and other large data sources.”

### Social Network Analysis and Agent-Based Modeling

The synergy between health science and computational methods clearly goes both directions. Working in the context of health-related issues raises interesting technical challenges that, in turn, may lead to valuable contributions in terms of algorithms and computational methods.

For example, most research about health and social media has focused on the content of social media. Yet, perhaps the true value of social media is the underlying structure of the social networks they create. It is theorized in interpersonal health behavior models that individual perceptions and behavior are significantly influenced by that individual’s social network (e.g., family, friends, community) [4, 38]. To leverage a user’s community, or social circle, one needs a method to extract it from social media platforms. One such platform, as mentioned above, is Twitter. There are significant challenges with Twitter in this context: (1) the underlying Twitter network is too large and too dynamic to be known or processed; (2) relations on Twitter, unlike Facebook for example, are directed, i.e., one user may follow another with no enforced nor expected reciprocity; and (3) an individual may belong to several overlapping social circles (e.g., work department, sport club, neighborhood reading group). While there has been work in community mining within computer science (e.g., see [23] for an excellent survey), very few, if any, have addressed all of these issues. An algorithm was recently designed and implemented to fill that gap [9]. Of significance here is that, had it not been for the fact that work was being done on health-related issues, where relationships are important, and within the context of Twitter, where relations are directed, the authors would probably not have thought of designing such an algorithm.

A similar synergy exists with agent-based modeling, where health-related issues may lead to the design of custom models (e.g., see [41]), and agent-based models may be leveraged to address complex, systems level issues (e.g., see [5, 50]), or applications where interactions or co-locations play a role, such as drinking behavior (e.g., see [24]) and disease spread (e.g., see [18, 46]). A recent article provides a brief overview of agent-based modeling, highlights a number of examples of their applications in the context of chronic diseases, and offers some thoughts on future research directions [37].

### Human-Computer Interaction

One of the other advantages of CHS is that it makes it possible to leverage the strengths of both humans and computers. While some may see this as a threat, we consider it an opportunity, one in which computational techniques complement and enhance human expertise, and where humans are not replaced, but enabled to move up the value chain.

Zamith and Lewis [54] argue in the context of coding that “an algorithmic approach departs from traditional content analysis in that it can generally be scaled up with ease… Researchers may thus use a larger sample, which is generally more likely to represent the overall population.” On the other hand, they recognize that “while algorithmic approaches yield satisfactory results in surface-level analyses or analyses that focus on structural features, their performance is significantly worse when assessing more complex features of texts.” Thus, in the spirit of CHS, they advocate a hybrid approach, where unique human and computer strengths are leveraged: “The development of computational tools and frameworks that can facilitate the blending of human judgment and algorithmic efficiency strikes us as an area of research that deserves additional attention.” Again, given their interest in coding, they say that “a hybrid approach must be further developed, one that preserves the contextual sensitivity and validity that are central to traditional content analysis and combines it with the large-scale capacity and reliability of computational approaches… truly [blending] the best of both worlds, human and machine alike.”

### Other CHS Opportunities

There are, of course, many other application areas and health issues where interdisciplinary CHS research offers unique opportunities. We list a few examples below. Again, these are not intended to be exhaustive, but serve as illustration, based on our knowledge of the current state of research.

- The design of novel geo-location algorithms based on content and context to support the study of epidemics.
- The design of topic detection and modeling algorithms when text content is sparse (as on Twitter).
- The combination of expert-driven topic selection with unsupervised machine learning to analyze health issues.
- The analysis of social networks and their impact on health behaviors.
- The study of online machine learning techniques and low-energy devices for human activity monitoring (e.g., quantified self).
- The integration of survey and data mining technology (e.g., MTurk and Twitter) to improve predictive models of risk behaviors.

In addition, increased use of mobile communication devices linked to the Internet and social media applications has led not only to a digital revolution but also to new health care innovations. mHealth represents a new form of health care delivery and treatment where patients are able to interact with their health care providers through mobile devices—providing additional “bread crumbs” for studying/mining health behaviors and health outcomes [20].

## Learning to Think Together

One of the risks of interdisciplinary work, that may cause researchers to hesitate, is that contributions may become imbalanced in that only one of the disciplines benefits, while the other is merely used. We bring this up here, because, while, for example, *using* computational techniques to solve hard problems is regarded as a worthy pursuit in all other disciplines, it is generally not considered as *contributing* to computer science, and thus will not help in such things as tenure and promotion cases for computer science colleagues. Clearly, a similar problem may arise in the other direction where the discovery of novel algorithms, while remarkable for computer science, is of no value to health science unless these algorithms help address a substantive problem.

Now, we are certainly not suggesting that all health scientists and computer scientists should work on CHS. But for computer scientists who have an interest in health, and for health scientists who have an interest in computer science, yet hesitate to engage each other, we wish to argue that such a collaboration is possible and can be fruitful for and rewarding to both disciplines. New and exciting areas of research, such as bioinformatics, which started with biology and computer science, stand as a testament to the great potential of doing research at the boundary of disciplines [30].

However, to quote a well-known result from machine learning, one of the areas of computer science that has showed great relevance to CHS, *there is no free lunch*. In order for a truly interdisciplinary approach to reap the benefits, both groups must be willing to:

- entertain different views of the world,
- appreciate the value of different yet complementary research methods,
- develop a shared vocabulary,
- learn to communicate their ideas effectively, and
- negotiate the publication of research to reflect the journals of each discipline to the benefit all.

Indeed, when interdisciplinary groups are willing to exercise these ideas, real team learning occurs, where “thinking is done together” [49]. Participants begin to share their experience, insights, understandings, and skills regarding novel research questions and methods.

Team learning requires commitment, discipline, and a sustained effort through time. Hindman [29] argues that “if social scientists are to do better at model building and quantifying uncertainty, they must broaden their horizons and adopt methods and practices from computer science and computational statistics. That requires some basic familiarity with machine learning algorithms beyond the linear models that are overwhelmingly dominant in social science today.” On the other hand, however, he also emphasizes the importance of domain knowledge, claiming—and rightly so—that “it is flatly untrue that successful data mining does not require substantive knowledge.”

Regularly scheduled meetings, ideally every week, create the opportunity to update one another on existing projects and to consider new opportunities provide the necessary scaffolding to build successful interdisciplinary CHS groups. Discussing the challenges and questions of existing projects allows students from the various disciplines to understand new perspectives, and faculty to learn new approaches beyond the typical boundaries of their formal training. Students can rehearse what they have recently learned in a class or a project with CHS colleagues both to share new insights and to reinforce what they have learned. Meetings also provide a time where participants develop and practice their shared vocabulary as discipline-specific approaches and methods are presented and discussed. For example, a health scientist may present a popular behavior theory used in practice, while a computer scientist may expose the algorithmic details of a machine learning approach of interest. These sharing sessions thus provide a safe and effective environment for discipline-trained faculty to develop new tools and skills outside of their field of primary expertise later in their career. They can in turn be particularly helpful to consider new ideas and innovative approaches to current questions, that could not have been as readily anticipated in typical multidisciplinary approaches.

Sharing questions and needs is enhanced by also understanding the shared and unique research approaches of each discipline. Computer science and health science have complementary “ways of knowing,” or methodological and teleological approaches. In order to effectively integrate, it is necessary to develop a shared understanding (and vocabulary) for how knowledge is built, from each side of the aisle. While some subject matter expertise is essential to forming the relevant research questions, it is necessary to develop a shared understanding of research methods and ways of establishing valid findings. Learning to explicitly share the conceptual foundations of each discipline (health/social science and computer science) can be expected to produce robust CHS. For example, there are established qualitative methods for developing knowledge and anchoring human interpretation in a conceptual framework that prevents subjective judgment from biasing interpretations. Similarly, quantitative social science/statistical methods provide established frameworks for making sound inferences. Both qualitative and quantitative social science have foundational principles in research design.

We have tried to illustrate from our own experience and that from others how CHS can foster a fruitful interdisciplinary approach, with the goal of developing algorithms and technology to better understand human interactions and enhance supporting environments by conducting innovative research that will inform future practice directed at changing health behavior through improved surveillance, communication, networking, and targeted intervention.

## Contributions to the Special Issue

The goal of this Special Issue on Computational Health Science is to begin our exploration of some of the specific opportunities offered by social media and sensor data to inform future practice directed at changing health behavior through improved surveillance, communication, promotion, and targeted intervention. Following our Call for Papers, we received a total of seven submissions. Each submission was reviewed by at least three referees for its relevance to the Special Issue, as well as its adherence to the same technical criteria used for all submissions to the *Journal of Health Informatics Research*. The four articles that follow were selected to be part of this Special Issue. While they clearly do not cover all aspects of Computational Health Science, they offer a sample of some interesting opportunities and relevant issues.

### Timing of Coping Instruction Presentation for Real-time Acute Stress Management: Potential Implications for Improved Surgical Performance

In their paper, Kennedy and Parker focus on the ever-present issue of stress in complex healthcare tasks and consider the impact of brief coping instructions at physiologically relevant times on indicators of physiological stress, such as heart rate variability (HRV). While the experimental context considered is that of a first-person shooter computer game, it provides a controlled environment to observe meaningful and relevant stress conditions, as would arise in many typical healthcare tasks. The results suggest that coping instructions received at times of elevated heart rate lead to better recovery from the stressor than do not receiving coping instructions, or receiving such instructions at non-physiologically relevant times. There also seems to be some evidence that intervention adherence yields better performance on acutely stressful tasks, and that such adherence is more successful when the user already has some mastery of the tasks. Although preliminary, these results are instructive and, given the necessity of maintaining high levels of performance under stress, warrant further evaluations in actual or simulated healthcare settings.

### Correlating Multi-dimensional Oculometrics with Cognitive Performance in Healthy Youth Athletes

Pradhan et al. describe a method to identify mild changes in cognitive performance in order to detect early signs of concussion in young people. The authors carry out eye-tracking studies on 440 American football players and cheerleaders aged 7 to 15 years in Arizona, testing them using multidimensional oculometrics, namely fixations and saccades. A key feature of the work is that youth of different ages are tested on different tasks, some effortful, as fixation and saccade patterns are known to vary according to age and required cognitive load. The results show that an approach utilizing fixation time, saccadic velocities, and saccadic amplitudes provides insight into cognitive performance across the age range. The work is conceptualized as a first step towards the development of a validated measure and reference data set for the rapid identification of mild cognitive impairment across the age range. The hope is that eye-tracking data can be used in the future as an objective index for changes in cognitive ability after head trauma.

### Validity of Consumer Activity Wristbands and Wearable EEG for Measuring Overall Sleep Parameters and Sleep Structure in Free-Living Conditions

Liang and Chapa Martell study the accuracy and validity of two sleep monitoring consumer products, namely the Fitbit Charge 2 wristband and the Neuroon wearable EEG-based eye mask. Given the importance of the quality of sleep in a number of different health conditions, as well as the increased access to and general interest in self-tracking, assessing the validity of consumer devices in this area is critical. For their study, the authors collect sleep statistics with each device for 25 subjects and compare them to a clinical gold standard given by the Sleep Scope EEG monitor. Interestingly, each device seems to have its own strengths, with the Fitbit wristband doing well at measuring the number of awakenings, and the Neuroon eye mask with good signal quality producing accurate results on total awake time and sleep onset latency. Neither device is able to measure sleep stage transition accurately. These results suggest that while consumer technology for sleep tracking may be adequate for general purpose and non-clinical use, it is still lacking in terms of diagnosing sleep disorders, and further advances are needed for trusted clinical use.

### Machine Learning and Mobile Health Monitoring Platforms: a Case Study on Research and Implementation Challenges

Boursalie et al. look at some of the specific challenges faced by the design, development, and deployment of machine learning-based health monitoring systems on mobile devices. The authors use cardiovascular disease monitoring as their case study. While the development of such a system is not novel, the organization of the case study around four main steps, namely data collection, data processing, model building using machine learning, and model deployment, along with recommendations for each, is insightful and should prove useful to both researchers and practitioners wishing to develop similar systems. In particular, the authors advocate and suggest methodological approaches, for 1) automatic data annotation to support predictive modeling, 2) data fusion, 3) multi-criteria model evaluation including both accuracy and efficiency (e.g., power consumption, execution time, CPU, and memory usage), and 4) visualization of accuracy/efficiency trade-offs to support improved decision-making.

## Conclusion

We have advocated the value of interdisciplinary research between computational and health sciences, highlighting both the associated advantages and inherent risks. We have argued that it is possible to develop a common research program where all disciplines are adequately served, and the result of the combined efforts is greater than the sum of its parts. There is clearly a host of interesting open research questions that CHS is uniquely positioned to address such as self-training (i.e., building training sets), identifying rare but critical events, improving record linkage across platforms, improving prediction with text and social relationships, mining multi-media content mining, and multi-language support. The papers selected for this Special Issue highlights some of these.

Interestingly, the National Science Foundation and the National Institutes of Health have recently joined to create a cross-agency research program, known as Smart and Connected Health. Similarly, the Robert Wood Johnson Foundation has launched its Health Data for Action initiative, and Kaggle continues to host competitions that explicitly draw on both computational and health expertise (e.g., Data Science Bowl 2017).

Based on our own experience as well as that of others, we offer the following as recommendations to enhance the practice of interdisciplinary work:

- *Vision*: Establish a common or shared vision and strategic direction that includes a desire to address real-world challenges using computational techniques.
- *Students*: Involve both undergraduate and graduate students who are typically millennials and motivated to address social issues.
- *Communication*: Determine a communication system that keeps the group connected and moving projects forward. Be patient and systematic in learning the language and culture of other disciplines.
- *Team*: Have a team-building attitude where team members are valued and encouraged by strong leaders and mentors.
- *System*: Challenge the system which is traditionally not supportive of interdisciplinary work. Create courses, degrees, and even tenure criteria that are supportive.
- *Win-win*: Creating a plan that supports the unique disciplinary scholarship expectations will help strengthen a long-term interdisciplinary framework for faculty and students alike.
